# Maxillary mucocele-A missed out 

**DOI:** 10.22088/cjim.14.3.577

**Published:** 2023

**Authors:** Sumitha Ramanadhan

**Affiliations:** 1 SreeBalaji Medical College and Hospital, Tamil Nadu, India

**Keywords:** Maxillary sinus, Mucocele, Marsupialisation

## Abstract

**Background:**

Maxillary sinus mucocele is a rare form of mucocele and are usually under diagnosed due to its vague symptomatic presentation. It is caused by obstruction of the natural ostium and accumulation of secretions inside the sinus cavities. It is a locally expansile lesion and symptoms are due to pressure on surrounding structures.

**Case Presentation:**

A 45 -year- old female patient presented with swelling on the left side of the face for 6 months with left infra orbital pain. On examination the swelling was diffuse in the left side of cheek. CT scan showed a homogenous opacity completely filling the maxillary sinus with expansion of the walls which helped in diagnosing the condition. Endoscopic marsupialisation was done and the patient is under follow-up for more than a year with no recurrence.

**Conclusion:**

Maxillary sinus mucocele is an epithelium lined sac filled with mucous secretions. They are expansile and can cause bony erosion of surrounding anatomical structures. It is mainly differentiated radiologically by the presence of air in the sinus cavity. CT scan shows homogenous opacity completely filling the antrum with no air shadow. The walls may be thickened or thinned out. Endoscopic marsupialisation of the mucocele gives excellent results with minimal recurrence. Maxillary mucocele being a rare benign cystic lesion is mostly under diagnosed. Hence, proper clinical examination and radiological evaluation help in early diagnosis. Appropriate surgical management gives a good success rate with nil recurrence.

Para nasal sinuses are empty spaces within the skull and are lined by ciliated columnar epithelium with numerous seromucinous glands. The secretions within the sinuses drain into the nose through the natural ostium. Mucocele are mucous containing cystic lesion which are formed due to blockage of the natural ostium with accumulation of secretions within the corresponding sinus cavities. When the secretions keep on accumulating over a period of time, it gradually enlarges which ultimately leads to erosion and the surrounding bone gets remodelled. They typically arise from chronic inflammation, scarring or surgical manipulation resulting in obstruction of the sinus ostia, although a compartment of a septated sinus can similarly become obstructed (1). Mucocele of maxillary sinus are very rare that etiology of such cases is either an untreated trauma or history of previous of sinus surgery (2). Maxillary mucocele if and undiagnosed and untreated can cause expansion of the sinus, bony wall resorption, and ultimately it extends into the adjacent bone structures. The mucocele can also cause orbital displacement, proptosis, diplopia, opthalmoplegia, and visual complications (3). In One third of the cases it is idiopathic in origin. The mucocele expands locally causing thinning of the sinus walls with extension into orbit and intracranium. Fronto ethmoidal mucocoele are common accounting for 89% of mucocele of the para nasal sinuses and the least common are maxillary mucocele. 

Though mucocele being a benign lesion, its expansion into the surrounding anatomical structures can cause severe and permanent complications and damage if they left undiagnosed and untreated. Thus prompt diagnosis and suitable treatment methods are very important. Thus the current case study involves in reporting the unusual maxillary mucocele found in a 45-year-old female. The diagnosis treatment method followed for that particular patient is mentioned in detail for the future use.

## Case Presentation

**Figure 1:**
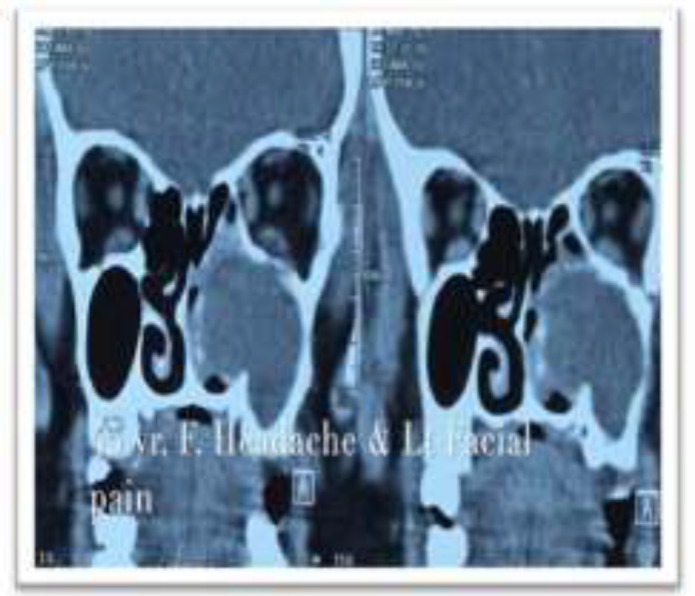
CT scan showing homogenous opacity with no air shadow and thinning of the infra orbital margin

A 45-year-old female was presented with facial swelling on the left side of face for 6 months with pain in the left infra orbital margin for the past 2 months. Swelling was progressively increasing in size without pain initially. Patient started getting pain later which gradually worsened. Patient had no history of recurrent upper respiratory tract infection, fever and headache. There was no history of blurring of vision or diplopia. Patient has been on treatment with a GP for 2 months for swelling and pain but had no improvement. On clinical examination, the swelling was diffuse in the left side of cheek with normal skin. There was thinning of the infra orbital margin with tenderness in the medial aspect of the infra orbital rim. Diagnostic nasal endoscopy revealed a smooth bulge in the inferior and middle meatus. There was no evidence of infection or discharge in the middle meatus and the mucosa was normal. CT scan showed a homogenous opacity completely filling the maxillary sinus with expansion of the walls which is shown in figure 1. 

**Figure 2:**
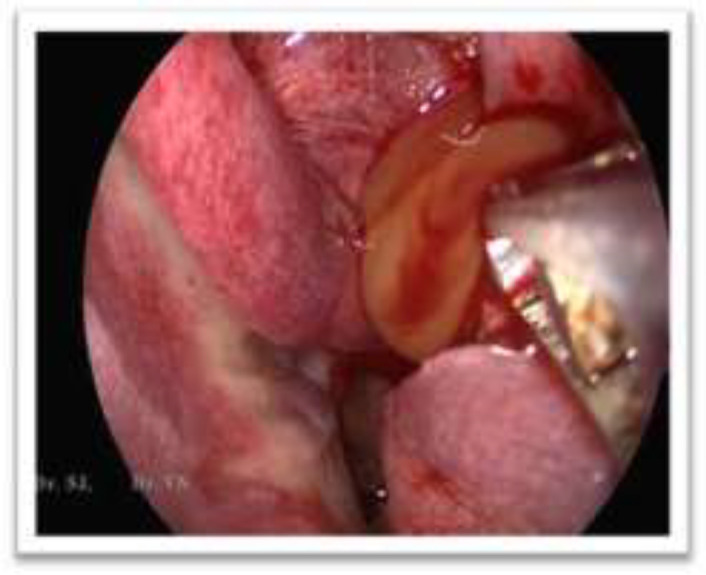
Draining out of the mucocoele contents

**Figure 3:**
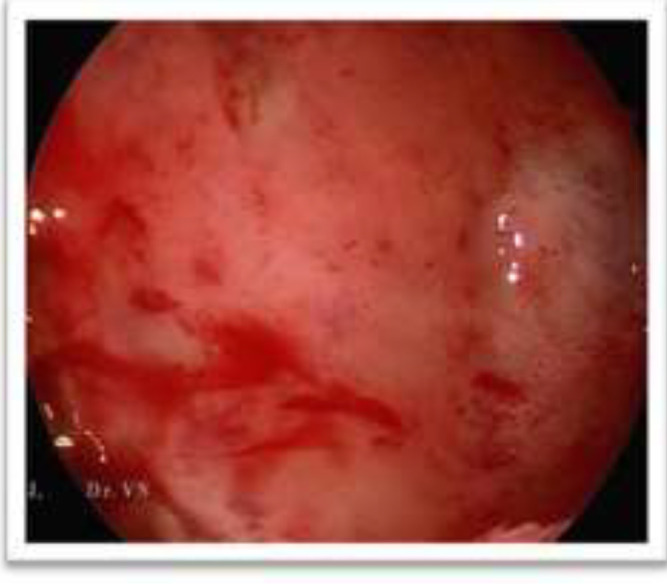
Post operation maxillary Antrum through the widened ostium after 4 weeks showing normal mucosa

**Figure 4:**
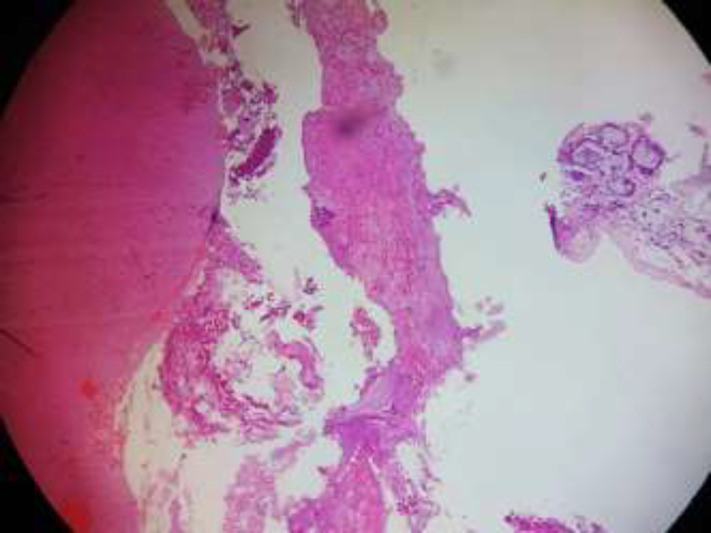
Histopathology of the lesion showing nasal cavity lined by stratified squamous epithelium, filled with fibrinous inflammatory exudates and desquamated cellular debris

The infra orbital margin was thinned out and elevated extending into the orbit. All other sinuses were normal. Visual acquity and field of vision were normal. Endoscopic marsupialisation of the mucocele was planned. Using nasal endoscope, a wide bore needle was inserted through the smooth bulge in the inferior meatus and 10 ml of straw-coloured fluid aspirated. The apiration of the contents of the mucocele is shown in figure 2. This reduced the bulge in the middle meatus and the anatomy of the infundibulum was clearly visualized. Middle meatal antrostomy was done and the entire content was drained out. The mucocoele was exteriorised into the nasal cavity. The infra orbital margin was palpated. It was thinned out but there was no defect palpated. Post operation period was uneventful and patient was relieved of the pain immediately. Figure 3 shows the post operation maxillary antrum through the widened ostium after 4 weeks which shows normal mucosa. Patient is on regular follow-up for the past 1 year with no recurrence. The antral and nasal mocosa along with the lining of the mucocoele was sent for histopathological examination (figure 4).

## Discussion

Maxillary sinus mucocele is an epithelium lined sac filled with mucous secretions. It is very common in ethmoid and frontal sinuses and rare in sphenoid and maxillary sinus. It is mainly due to blockage of the natural ostium. Obstruction of the sinus ostium has been suggested as the primary etiologic factor. This may be due to a mass lesion, inflammation and fibrosis, osteoma, fibrous dysplasia, Paget's disease, malignancy, trauma, or previous surgery (4). In some cases, it is idiopathic with no obvious etiological process.  They are expansile and can cause bony erosion of surrounding anatomical structures. Therefore, they must be removed or drained to prevent intracranial and orbital extension (5). Expansion occurs due to the direct effect of increasing positive pressure within the mucocele. Local production of bone resorption factors such as prostaglandins, interleukin-1, and tumor necrosis factor have also been identified at the interface between the mucocele and bone. These may cause intraorbital or intracranial extension (6-8). Since the mucocele are non-infectious they are usually asymptomatic. They are sterile and painless with pain indicating infection (9). Patient starts getting symptoms when it extends into surrounding structures by local expansion or gets infected forming pyocele which causes pain and other constitutional symptoms. This diagnosis is easily missed as the patient presents very late due to under diagnosis. In our case, patient presented with infra orbital and facial pain after it started encroaching the orbit. Most common differential diagnosis is maxillary retention cyst. It is mainly differentiated radiologically by the presence of air in the sinus cavity. CT scan shows homogenous opacity completely filling the antrum with no air shadow. The walls may be thickened or thinned out. Endoscopic marsupialisation of the mucocele gives excellent results with minimal recurrence. It does not interfere with the mucociliary mechanism of the sinus and so the secretion, drainage and ventilation of the sinus is unaffected. There is no external scar and recovery is very quick. The maxillary sinus is widened and the lining of the mucocele is exteriorized to the nasal cavity. The recurrence rate is ~1% with marsupialization (10). Martel et al. analyzed 58 patients of paranasal sinus mucoceles and found that recurrence rate was lesser in patients treated endoscopically (4.8%) than those treated by an external approach (28.5%) (9).

Maxillary mucocele is a benign cystic lesion occurring due to blockage of natural ostium. It causes expansion of sinus walls with extension into the surrounding anatomical structures. Since it is a rare entity, it is usually missed out during diagnosis leading to unnecessary investigations and procedures. Clinical examination and radiological evaluation help in early diagnosis. Appropriate surgical management gives a good success rate with nil recurrence.
